# Opal: An open source ray-tracing propagation simulator for electromagnetic characterization

**DOI:** 10.1371/journal.pone.0260060

**Published:** 2021-11-17

**Authors:** Esteban Egea-Lopez, Jose Maria Molina-Garcia-Pardo, Martine Lienard, Pierre Degauque

**Affiliations:** 1 Dpt. Information Technologies and Communications, Universidad Politécnica de Cartagena (UPCT), Cartagena, Murcia, Spain; 2 Institute of Electronics, Microelectronics and Nanotechnology (IEMN), University of Lille, Lille, Villeneuve d’Ascq, France; Norfolk State University, UNITED STATES

## Abstract

Accurate characterization and simulation of electromagnetic propagation can be obtained by ray-tracing methods, which are based on a high frequency approximation to the Maxwell equations and describe the propagating field as a set of propagating rays, reflecting, diffracting and scattering over environment elements. However, this approach has been usually too computationally costly to be used in large and dynamic scenarios, but this situation is changing thanks the increasing availability of efficient ray-tracing libraries for graphical processing units. In this paper we present Opal, an electromagnetic propagation simulation tool implemented with ray-tracing on graphical processing units, which is part of the Veneris framework. Opal can be used as a stand-alone ray-tracing simulator, but its main strength lies in its integration with the game engine, which allows to generate customized 3D environments quickly and intuitively. We describe its most relevant features and provide implementation details, highlighting the different simulation types it supports and its extension possibilites. We provide application examples and validate the simulation on demanding scenarios, such as tunnels, where we compare the results with theoretical solutions and further discuss the tradeoffs between the simulation types and its performance.

## Introduction

Accurate characterization and simulation of electromagnetic (EM) propagation has been a key ingredient of electrical engineering and research for the last decades [1]. Within the available methods [1], ray-tracing (RT) methods provide very high precision, usually at the cost of demanding computational power and a strong dependence on the quality of the tri-dimensional (3D) model of the environment [2]. RT methods are based on a high frequency approximation (optical ray) to the Maxwell equations and describe the propagating field as a set of propagating rays, reflecting, diffracting and scattering over environment elements. As a result, this approach has been usually too computationally costly to be used in large and, especially, *dynamic* scenarios [3], and so networks simulators usually only provide stochastic or simplified hybrid methods [3].

But RT is becoming increasingly more important for the evaluation of wireless systems: its necessity is already recognized for current cellular systems (5G) [4], but especially for next generation cellular networks [5], which incorporate the use of visible light communications and techniques such as adaptive beamforming and spectrum management and other advanced transmission mechanisms, whose performance is not captured by just computing signal-to-noise ratio but require a multidimensional assessment of the channel. RT can provide it and, in fact, the behaviour of high frequency signals matches better the ray-optics approximation and increases the influence of multipath contributions from environmental elements. Simultaneously, the computational power of graphics processing units (GPU) has been leveraged to simulate propagation [2, 6] at a much more reasonable computational cost. However, RT still requires as a previous step the capability to build 3D models of the scenario [2, 3] but current simulators do not usually provide it. And despite the availability of open real-world map data [7], tools to convert it to 3D scenes suitable for propagation simulation are scarce.

To address those shortcomings, in a previous paper [8] we introduced Veneris, an open source framework made of a traffic simulator, implemented on top of the Unity game engine [9], a ray-tracing GPU based propagation simulator, called Opal, and a set of modules which enable bidirectional coupling with the widely used OMNET++ network simulator [10]. With Veneris we leveraged game engines to provide simulators with a physics engine and capabilities for general-purpose 3D representation which allows to seamlessly bring 3D models. Since then, we have extended Opal with the capability to use multiple methods of propagation simulation, curved environment elements, and the rendering of the propagation paths. Opal can be used as a stand-alone RT simulator, but its main strength lies in its integration with the game engine, which allows to generate customized 3D environments quickly and intuitively, usually from OpenStreetMap (OSM) data [7], but also by taking advantage of the programmability of the game engine.

Despite the remarkable number of works that use RT techniques and the steady introduction of new methods [11], there is an evident lack of available open source tools and libraries. Most of the research software is not released or is only commercially licensed. Several open source simulators that do not use RT are available, such as SPLAT! (https://www.qsl.net/kd2bd/splat.html) and Radio Mobile (http://www.ve2dbe.com/), which are radio frequency signal propagation simulators which includes terrain losses. Many of the related open source tools use Matlab (https://www.mathworks.com) as computation engine, which requires a license. For instance, NYUSIM (https://wireless.engineering.nyu.edu/nyusim-5g-and-6g/) provides custom channel models for 5G and 6G simulation and SimRIS (https://corelab.ku.edu.tr/tools/simris/) supports reconfigurable intelligent surfaces. Regarding currently available open source tools with support for RT we can only find RadioPropa (https://github.com/nu-radio/RadioPropa) for a specific application, propagation in inhomogeneous media, and a general-purpose wireless simulator, PyLayers (https://pylayers.github.io/pylayers/), which provides some functionality similar to Opal, but lacks other, such as environment generation tools. In fact, to the best of our knowledge, integrated tools with 3D environment generation and propagation simulation are only commercially available. For instance, among the proprietary commercial tools that include at least a similar set of features, including moving elements and antenna radiation patterns, we can only find Altair Feko (https://www.altair.com/feko-applications/) or Siradel solutions (https://www.siradel.com/solutions/software/). Opal, as an open source, MIT-licensed and extendable framework may alleviate this gap. It is available for Linux and Windows operating systems at http://pcacribia.upct.es/veneris or from its public repository http://gitlab.com/esteban.egea/opal.

In this paper we describe its implementation and discuss its main features and current limitations. First, we provide a brief review of the Veneris framework. Then, we describe the main features of Opal and relevant implementation details. Afterwards, we provide application and validation examples which illustrate its capabilities as well as its main limitations and trade-offs. We conclude with a brief discussion of related works and future work.

## Background

**Fig 1:**
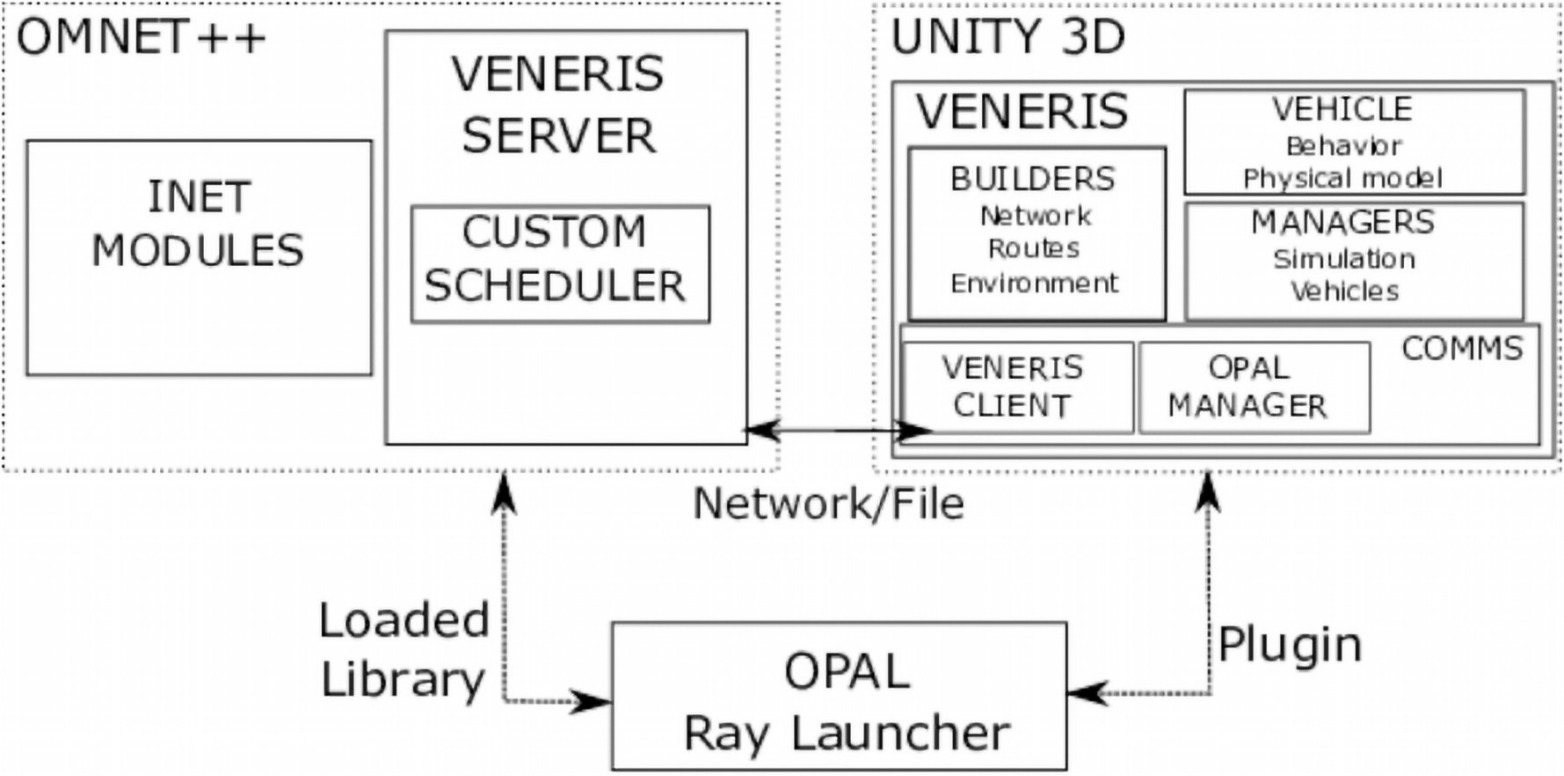
Veneris main components. Opal can be used as a standalone application, as a plugin with Veneris or loaded with OMNET++.

Opal is part of the Veneris framework. Although in this work we focus on its particular features, we first provide a brief overview of the overall framework to provide context. Parts of the description are taken from [8] where additional details can be found. Veneris is made of a set of tools that provide different functionality and can interact with each other. The main components, shown in Fig 1, are:

- **Veneris simulator**. A set of Unity [9] components that provide a realistic microscopic road network simulation in an interactive 3D environment. The components have been grouped in functional modules. Builder components are used to generate the scenario elements: roads, intersections, traffic lights or buildings. Vehicle components include a model of the dynamics of the vehicle and components which model the behavior of the vehicles on roads, intersections and the interaction with other vehicles. Communication components implement the communication with simulation modules. Managers handle different aspects of the simulation globally.
- **Opal**. The main focus of this paper, it is a ray-launching based, deterministic radio-frequency propagation simulator, implemented in C++ with NVIDIA OptiX [12], a library and application framework for high performance ray tracing on the GPU.
- **Veneris OMNET++ modules**. A set of OMNET++ and INET [10] modules which enable bidirectional coupling between the communication network and the traffic simulation.

These tools can be used independently or combined in different ways to perform different types of simulations. The main combinations are:

- *Traffic simulation*: a scenario from real-world map data is built and run with Veneris to obtain traffic-related metrics.
- *Hybrid network-traffic simulation with RT-based propagation*: the traffic simulation is combined with a network simulation with any protocol stack from OMNET++. Opal computes propagation by RT but any other propagation model from OMNET++ can be used instead.
- *Network simulation with ray-launching based propagation*: a OMNET++ simulation is run with RT-based propagation computed by Opal.
- *Electromagnetic characterization*: a 3D environment, tailor-made or generated from map data, is built with Veneris and a propagation simulation is launched with Opal.

**Fig 2:**
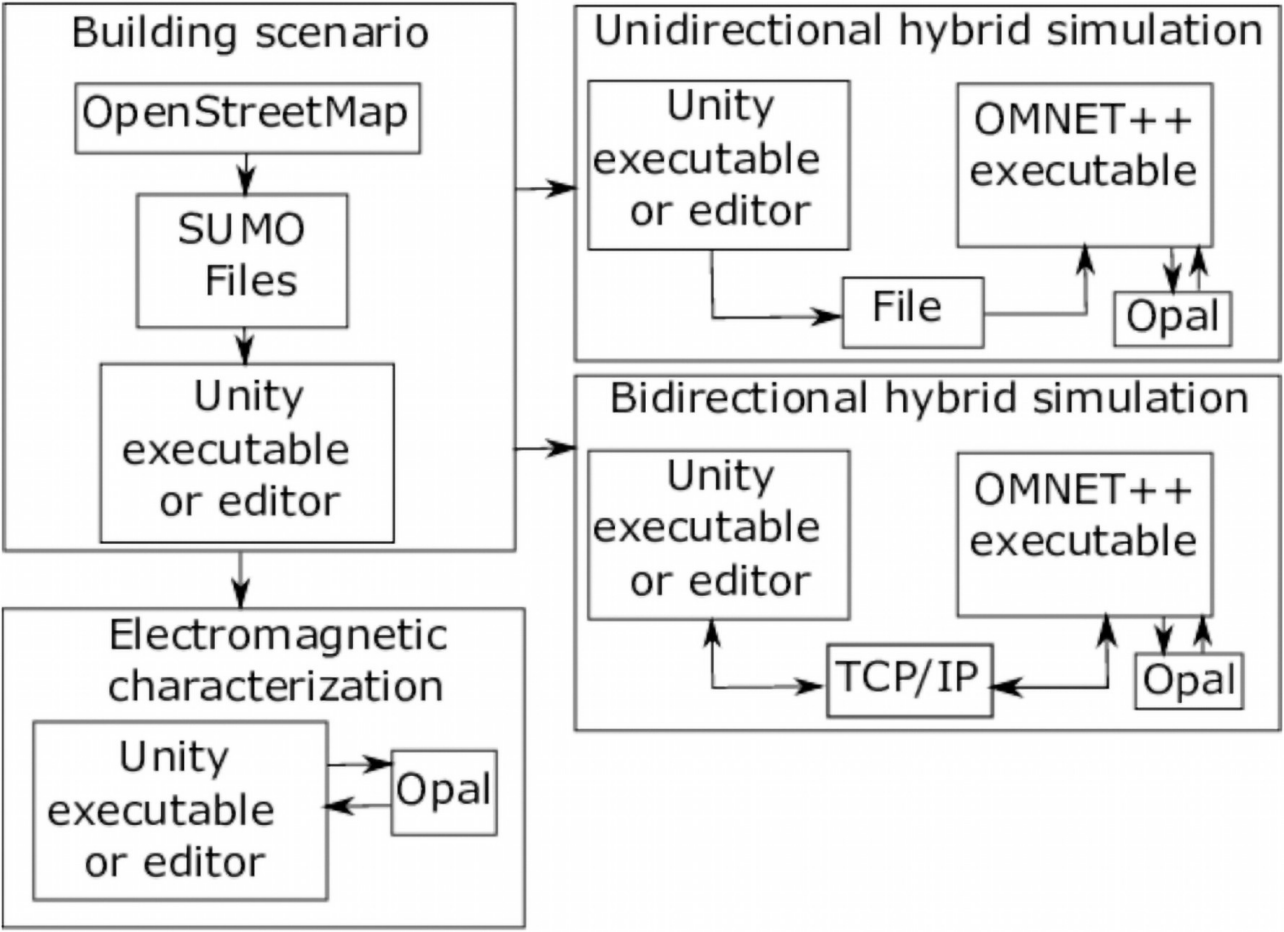
Workflow and types of simulations with Veneris and Opal. First, the scenario is built from real-world map data. Then, three alternatives are available: (1) to carry out the electromagnetic characterization of the scenario, just with Opal; (2) to run a unidirectional hybrid simulation, where the traffic simulation output is stored and then fed to the OMNET++ modules; (3) to run a bidirectional simulation, where traffic and network simulation are run simultaneously and interact with each other.

The usual workflow and simulation types is depicted schematically in Fig 2. In this paper we will discuss mainly using Opal for EM characterization, which can be run as a standalone command line application or from the Unity editor or executable. In the first case, Opal is typically used as a library, through a custom C++ program which incorporates calls to Opal functions to load meshes and launch the NVIDIA OptiX kernels which actually perform the RT. In the second case, a compiled library is used as a Plugin for Unity programs, via the interface provided by Opal.

## Opal

### Implementation

Opal uses the shooting and bouncing (SBR) method [2]: electromagnetic waves are simulated by rays launched from the transmitter, either with a given angular separation or with other methods as we will see. These rays are propagated along their trajectory until they hit an obstacle where they are reflected, diffracted, transmitted or scattered. Subsequent rays are traced again. The contributions of the different rays that hit a reception sphere around the receiver are added to compute the electric field.

Opal has been implemented in C++ and uses NVIDIA OptiX [12], a general-purpose GPU ray tracing engine for rendering. The programming model of OptiX involves a host-based (CPU) API used to define data structures for ray tracing and a device-based (GPU) CUDA API, used to generate rays, intersect them with surfaces and process those intersections. The engine calls a set of user-provided programs, the *generation*, *closest-hit*, *any-hit*, *intersection* and *miss* programs, when the corresponding events occur during the ray traversal. Therefore, the user of the OptiX API has to implement those programs, which are provided as CUDA files. When the GPU has dedicated RT cores, the triangle intersection is computed by the hardware which reduces remarkably the computation time. With OptiX a scene is represented as a graph that controls the traversals of rays through the scene [12]. Nodes are made of geometric objects, transforms and other data objects, such as acceleration structures, based on bounding volume hierarchies (BVH) [2], that speed up the traversal and intersections on the graphs.

**Fig 3:**
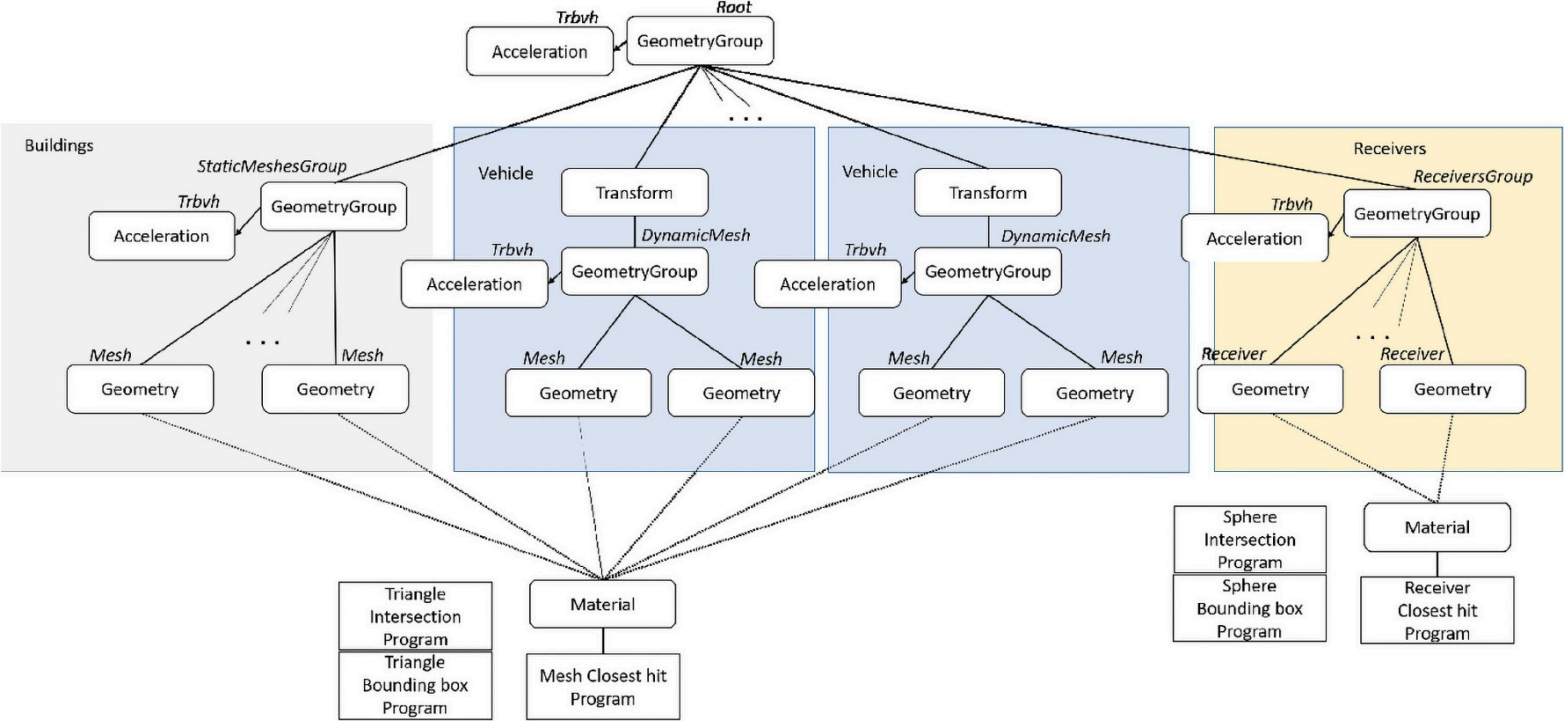
Opal scene graph. There are three groups in the graph: buildings or other static interactive environment elements, vehicles or any other moving interactive elements and receivers, which can be moved but since are made of spheres, do not need transformation matrices.

A defining feature of Opal is that works with both static and *moving* 3D scene objects, represented as triangle meshes. Objects, transmitters and receivers can be dynamically added and removed from the scene and subsequent ray launches take into account those changes. The structure of the scene created by Opal, shown in Fig 3, reflects this capability. All the static objects in the scene, such as buildings, share an acceleration structure below the root node and are assigned an intersection program, which computes ray-triangle (mesh) intersection, and one or more materials and a closest hit program. The receivers, represented as spheres, are attached just below the root node and also share an acceleration structure. When they move, their sphere coordinates are updated. Finally, the dynamical geometrical objects, such as moving vehicles, which may be made of a hierarchy of meshes, are assigned each one a transform node, updated when the object moves, and their own acceleration structure. Every mesh added to the scene has its own electrical properties, as defined by recommendation ITU P2040 (https://www.itu.int/rec/R-REC-P.2040/en), which are taken into account in the computations.

There are two main components in Opal: the OpalSceneManager and OpalSimulation classes. The former glues together the rest of components: is responsible for loading meshes in the device and creating the scene graph, holds receiver and transmitter managers, applies configuration options, provides results and holds the simulation classes. The latter is the base class for the different methods of simulation implemented. A new simulation is created by deriving from this base class. Let us remark that a simulation can be developed to implement a certain *propagation mechanism*, such as reflections, diffractions or scattering; or it can provide alternative *simulation methods* for the same kind of propagation mechanism. For instance, as explained below, reflections can be simulated with the basic or the ray density normalization methods. Opal is designed to handle multiple types of simulation whose execution will be called sequentially by OpalSceneManager. This allows us to combine or disable independent propagation mechanisms at will. As an example, we can add to a scene a n-order reflection simulation (the order refers to the maximum number of reflections allowed by the simulation; by n-order we mean that no particular limit has been imposed and, for instance, simulations with up to 60 reflections per ray can be seamlessly done) plus a single-order diffraction simulation. OpalSceneManager will execute all the simulations and combine the results. It can be executed again with diffraction disabled to examine the differences and so on. Of course, not all the simulation types can be run independently: a n-order reflection-diffraction simulation would require a specific simulation class, depending on the implementation. Simulations also include the set of CUDA files with the required OptiX programs such as ray generation, closest-hit, miss and so on. In general, the user program associated to the closest hit for meshes performs the operations required when a ray hits the geometry, such as computing the reflected ray, the reflection and/or diffraction coefficient so on. The closest hit program for receivers usually filter duplicates, depending on the type of simulation, and adds the ray contributions to the electric field. In this sense, let us remark, that in our implementation most of the required computations are done directly on the GPU when possible, including for instance, duplicate ray filtering, which is done using the Thrust library provided by CUDA [13]. Other approaches, such as [6], trace the rays and record the interactions and then post-process the logged trace to compute the field. While this procedure has its own advantages, it requires a huge amount of GPU memory, unlike ours, which also remarkably speeds up the simulations. Finally the generation program is responsible for creating the rays to be traced. In SBR methods, the full 3D unit sphere is discretized by some method and a ray per point is created. The number of rays generated per solid angle, that is, rays per stereorad (rays/sr), is the ray density of the simulation. Opal provides several methods described below.

As propagation mechanisms, Opal currently implements n-order reflections for flat, curved or combined flat-curved scenario elements and single-order diffraction as well as two alternative methods. They have been implemented with the following types of simulation:

- *Basic reflection (BR)*: A simulation of n-order reflection only for flat surfaces, where (1) both transmitter and receiver have the same polarization and (2) it is either purely vertical or purely horizontal. It is only marginally faster than full polarization simulations. Class BasicFlatMeshReflectionSimulation.
- *Linear polarization reflection (LPR)*: A simulation of n-order reflection only for flat surfaces where arbitrary linear polarization is used and computed. That is, transmitters and receivers can have arbitrary linear polarization and they can be different. Both this method and the previous one require duplicate-ray filtering. Class LPFlatMeshReflectionSimulation.
- *Ray Density Normalization (RDN)*: A simulation of n-order reflection for scenes with both curved and flat surfaces that uses the ray density normalization method [14]. RDN is a modified SBR method that launches a large number of rays that are uniformly distributed on space. All the rays that hit on the receiver sphere are added and normalized in order to get the correct field contribution. Let us note that this normalization is equivalent to a weighted average of the hit rays and so the result is approximation, though a very precise one usually. There are slighlty different ways to compute the normalization, called field and power trace [14], both of them implemented. With this method, thus, it is not necessary to filter out hits to get a single contribution for a given propagation path. This feature makes this method specially useful for simulation of reflections on curved surfaces, unlike the previous methods, which require some mechanism to decide how the rays are filtered with curved surfaces. If necessary, detailed field information can be stored on the GPU buffers but in the default mode the field contributions are added directly to the output buffer, which does not require memory reservation for hits on the GPU. This latter feature together with the avoidance of filtering allows Opal to launch simulations with an extremely high density of rays, such as 10^10^ rays/sr at the cost of longer simulation time. Class RayDensityNormalizationSimulation.
- *Single diffraction (SD)*. Single-order diffraction is computed using Unified Theory of Diffraction (UTD) [1] for all the diffracting edges in the scene. Let us notice how this is a different propagation mechanism, and so this simulation can be combined with any of the previous reflection simulations. On the contrary, the previous simulation types are alternative methods for simulating the same propagation mechanism, reflections, so one of them has to be selected for the simulation. Class SingleDiffraction.

In addition, we provide two experimental classes for curved surfaces that require duplicate-ray filtering and angle-discrimination but they are not reliable enough yet. In general scenarios with flat elements, all the methods provide similar results. For scenes with curved and flat surfaces RDN must be used. In scenarios with a high number of contributions from reflections, such as tunnels, and at long distances, RDN usually provides more accurate results, but, since it is an approximate method, in canonical scenarios such as a straight diffraction edge, it provides less precision. Let us remark that, even though RDN does not require duplicate filtering, the results do depend on the reception sphere radius. That is, all the above methods have a dependence on the reception sphere radius, which is a general problem of SBR methods, and so it is usually necessary to experiment with the radius parameter when evaluating scenarios. We exemplify these trade-offs in the Results section.

Finally, as output, by default Opal computes the induced voltage at the receiver antenna or, alternatively, the components of the received field at the receiver point. But with very little effort additional metrics can be computed, such as, Direction-of-Departure (DoD), Direction-of-Arrival (DoA), signal delays and so on, as required by the application.

### Additional features

To finish the implementation description, we summarize some other capabilities of Opal in the following paragraphs.

- *Multiple transmitters and receivers*. Opal can simulate in parallel multiple transmitters and receivers, with a notable performance gain compared to an equivalent sequential simulation. This feature is specially useful for electromagnetic characterization. A canonical example is the computation of cellular coverage in urban environments: hundreds of receiver points and several base stations can be simulated in a single run.
- *Multichannel*. Different frequencies can be simulated simultaneously. In the previous example, multiple transmitters using different frequencies can be simulated in parallel in a single run.
- *Duplicate ray filtering*. Multiple counting of received rays is a major problem of the ray-launching approach [15]. Removal of duplicates usually requires setting a receiver sphere radius dependent on the ray unfolded distance and keeping a log of the ray paths to track the sequence of hits and filter them in a post-processing phase, none of which is efficient in a parallel processing framework. We use a novel approach: each ray stores in a *single* integer the sequence of environment elements it hits, as a *combined hash* of the element face identifiers. Each time an element face is hit, the combined hash is updated. Since this hash is guaranteed to be unique for different sequences of faces, including permutations, it is used to remove all the rays following the same sequence except for the closest one to the receiver. This way, to filter duplicates it is only necessary to store just a single integer per ray instead of a list of face sequences. The filtering is done as follows: All hits on a receiver are kept in GPU memory and, after tracing, they are sorted by transmitter identifier (id), receiver id, hash and distance of hit ray to receiver, in this order. Duplicates are removed by considering equal all hits with the same transmitter and receiver id and hash, so that only the closest hits to the receiver with different hashes are kept. The operation is done directly on the GPU applying first the sort and then the unique functions of the Thrust library [13]. Notice that since all hits have still to be stored on GPU memory, a relatively scarce resource, *simulations that require filtering are usually memory-bounded*, which places a limit on the density of rays that can be traced. On the contrary, RDN does not have this limit and can use very high densities.
- *Moving objects*. By leveraging a ray tracing engine designed for high performance in interactive environments, transceivers and other objects can be added dynamically to the scene without performance degradation. Similarly, by updating their transform, complex mesh hierarchies are moved efficiently in the simulation.
- *Ray generation*. There is a great deal of flexibility for ray generation. First, the user can directly create a buffer with particular ray directions. Otherwise, rays can be generated using two different modes, *equispaced angular sampling (AS)*, where rays are separated by a constant azimuth and elevation angle; or *random uniform AS* where rays are generated randomly with a uniform distribution on a unit sphere solid angle. In addition, the generation range can be set to the desired values, as for instance, to isotropic (full sphere) which is the usual mode for most simulations when fast computation is necessary. But it can also be *sectorized*, where rays are distributed only on a certain solid angle for a given launch. It is used to increase the accuracy of the results, by scanning the scenario at consecutive steps (sectors) with a high ray density, like moving a beam of rays. The results for each sector are stored and merged at the end of the simulation. Finally, rays can be generated on CPU or on GPU directly at launch. The generation of rays at launch is much faster: as an example, when sectorized simulations are used, the simulation time is reduced 2 to 4 times. If rays have to be generated just once, there is no performance gain, but CPU generation requires using a buffer of rays, which means that for very high densities, that is, a very large number of rays per launch, a relevant fraction of the available GPU memory is used only to store ray directions.
- *Antenna patterns*. User can provide files with discretized antenna radiation patterns and Opal will apply the corresponding gain per ray according to the DoD at transmission and the DoA at reception. Gains can be selectively used with transmitters and receivers and applied independently at tranmission and reception.

## Results

In this section we describe and discuss some application examples of Opal. We first discuss the generation of scenarios and then four validation experiments.

### Scenario generation

**Fig 4:**
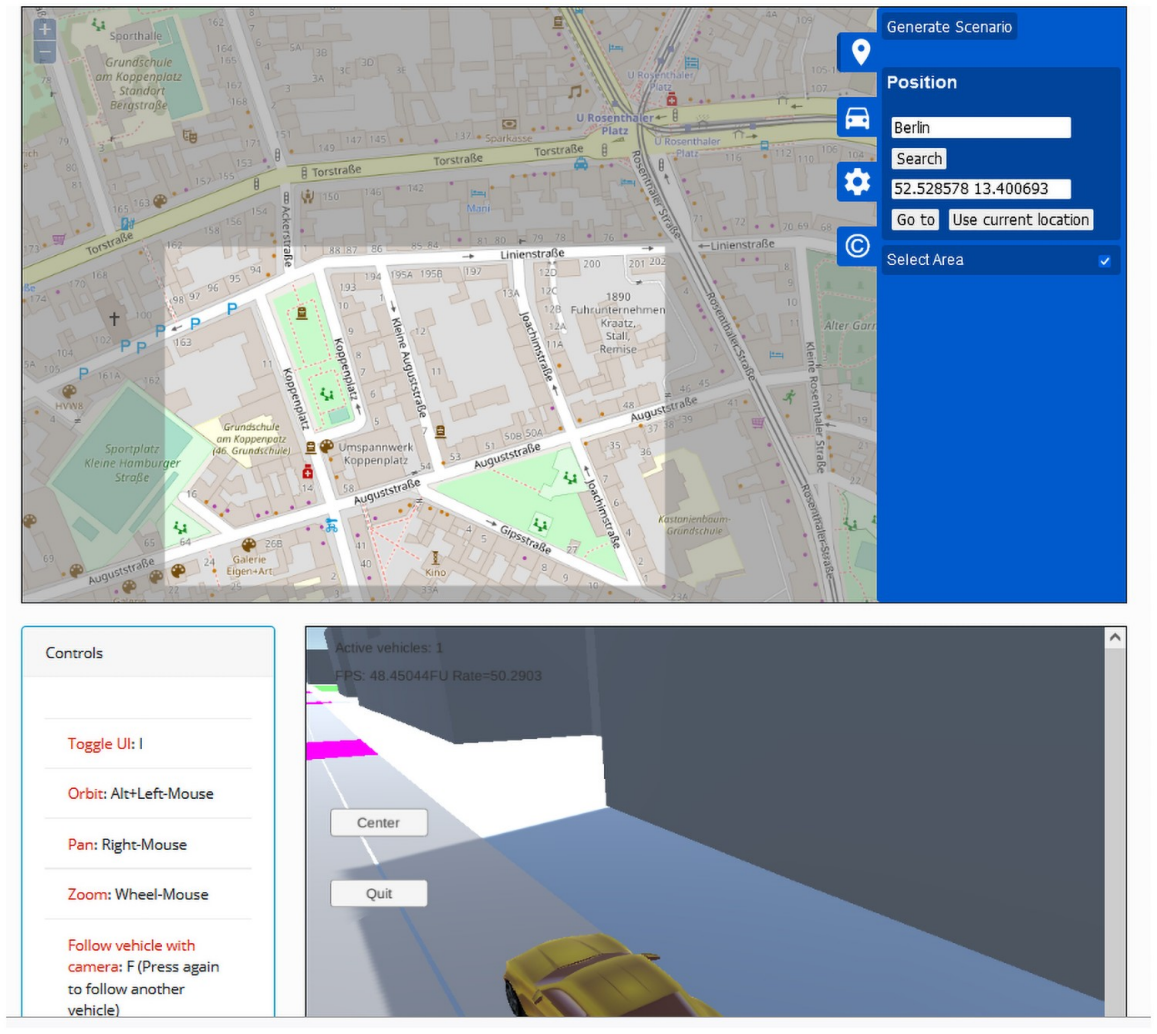
Web-based scenario generation. With a web interface the user selects an area from OSM and generates the scenario. The files can be downloaded but it can also be run directly on the browser, as shown in the figure, to check the results. Part of the Figure shows a map from OpenStreetMap: ©OpenStreetMap contributors.

**Fig 5:**
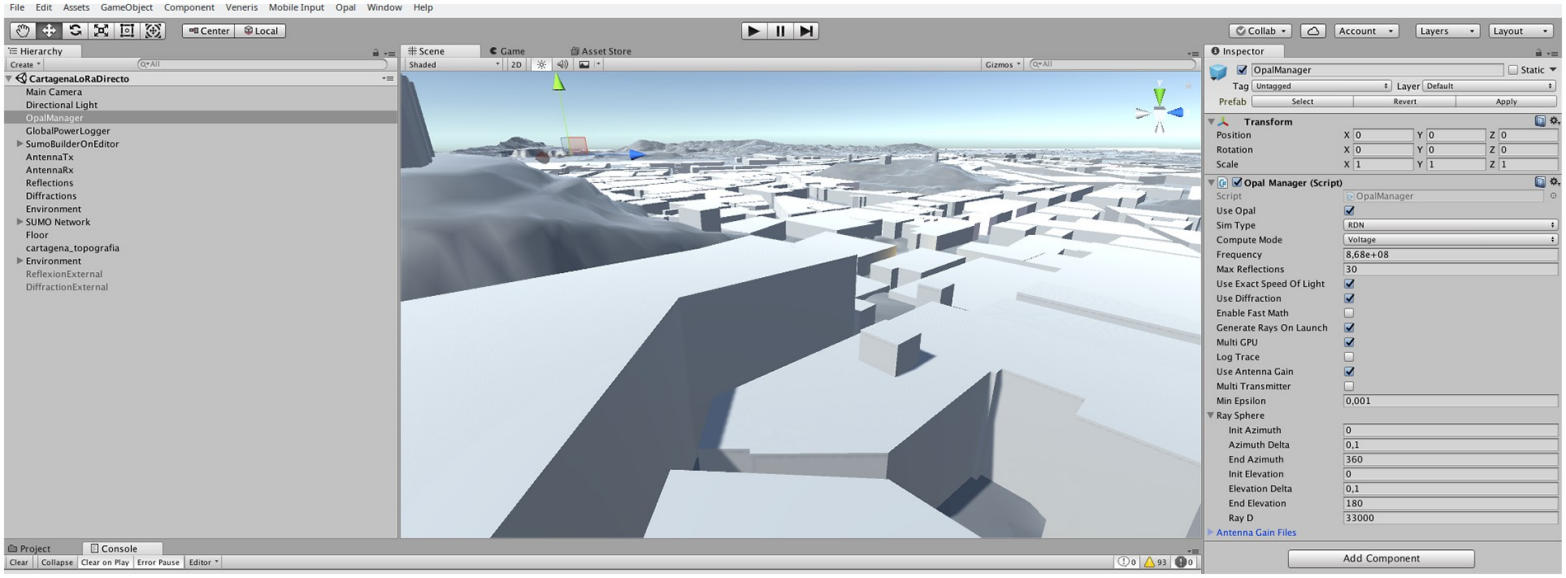
Cartagena scenario. To increase the simulation accuracy the Cartagena city scenario has been enhanced with terrain elevation data and high accuracy building data from public spatial information sources. Snapshot taken from the Opal tool.

Scenarios for EM characterization can be created with the automated generation tool of Veneris or by directly designing and/or programming the required elements. In the first case, Veneris can be used to generate traffic networks (roads and intersection), vehicle routes and the environment from SUMO files [8]. For EM, the network and vehicles need not be generated and only the environment is used, which is generated directly from OSM data. In fact, Veneris provides a web-based scenario generation tool (http://pcacribia.upct.es/veneris/scenario) with which the user just has to select the area of interest on the map and the scenario is generated for download. It can be even be tested directly on the browser as shown in Fig 4. Opal actually leverages SUMO to generate the element outlines and then downloads additional metadata from OSM, before elevating the elements and adding the required data structures for simulation: EM properties and diffraction edges among others. Let us note that this procedure is the minimum general way to construct automatically generated scenarios, but it depends on the quality of the OSM data, that can vary widely depending on the geographical area. Although for many purposes, such as vehicular simulation, the quality is enough, for more accurate simulations, it is usually necessary to enhance the base scenario. Therefore, additional data pipelines can be created. As an example, for our research, we have processed very accurate building data for the city of Cartagena, acquired from the Spanish Land Registry and we have also added terrain elevation data from the LiDAR database from the Spanish Instituto Geografico Nacional. A screenshot of the generated scenario is shown in Fig 5. The details depend on the particular spatial data source used, that should be processed and adapted to Veneris format.

**Fig 6:**
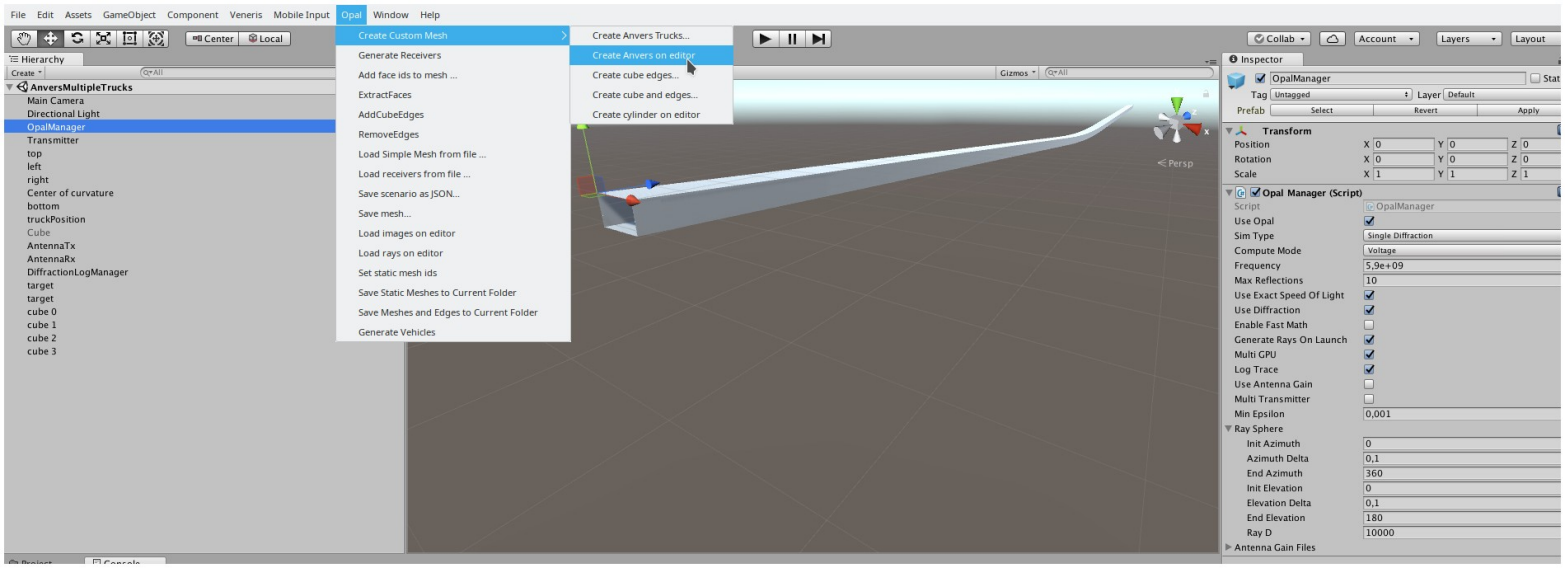
Anvers tunnel scenario. The tunnel has been procedurally generated and the Unity Editor menu has been extended to generate the scenario. Snapshot taken from the Opal tool.

In the second case, a custom scenario can be designed and built using the capabilities of the Unity engine. In addition to the basic 3D elements available, procedural generation can be implemented using the full power of the C# language provided by Unity, as well as its support for extending the Unity Editor with custom scripts. As an example, in Fig 6 we show the Anvers tunnel, where we have reproduced a real, slightly curved, underground road tunnel close to the city of Anvers. The tunnel curvature and dimensions can be accurately recreated with a C# script which can be added for future generation as a custom menu item in the Editor. In summary, these examples show the flexibility of Opal for scenario generation that can be achieved by combining both the provided scenario generation tool as well as the programmability capabilities of the Unity engine.

### Validation examples

In [8] we already validated Opal with, among others, a street-crossing and a city-wide simulation. In this work we provide new validation examples on scenarios that make use of the recently introduced features, such as curved surfaces and diffraction. The tunnel scenarios that we discuss next are actually more difficult to simulate than the outdoor urban simulation of [8] because of the influence of reflections on them. In fact, to obtain accurate results it is necessary to use a much higher ray density and raise the maximum number of reflections.

#### Rectangular tunnel

**Fig 7:**
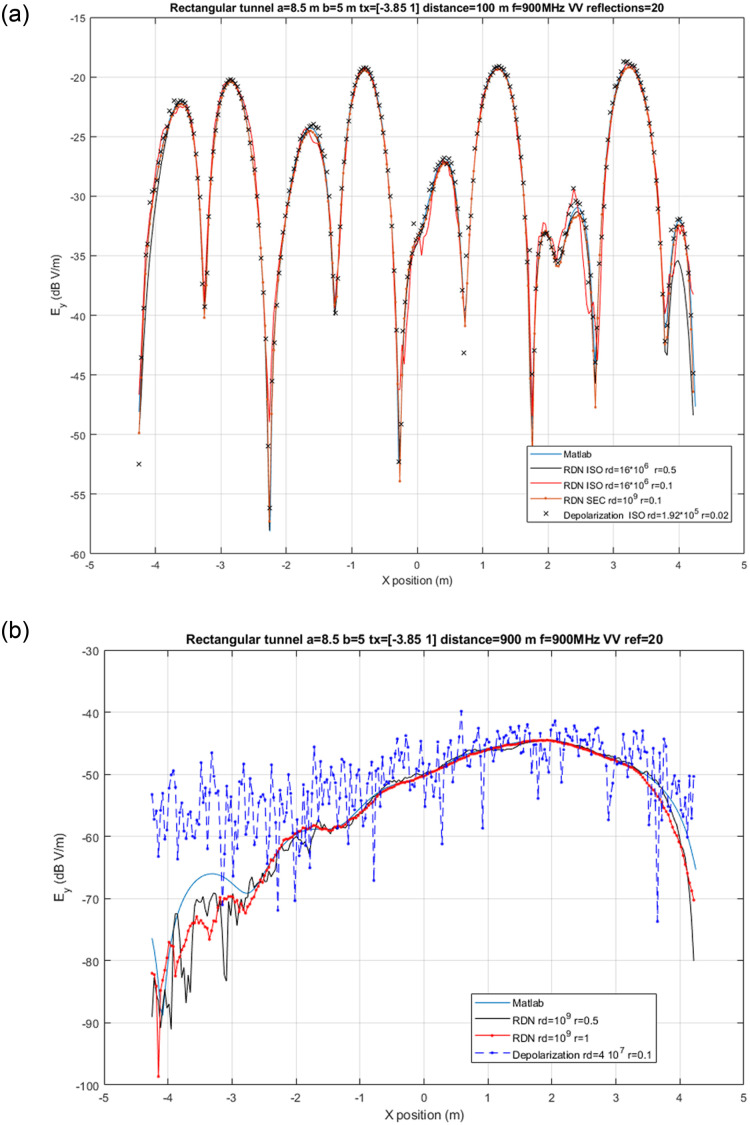
Rectangular tunnel. Simulation of received electric field at cross sections at z = 100 m and z = 900 m of a rectangular tunnel with dimensions 8.5m x 5m x 1000m, EM properties *ϵ*_*r*_ = 5 and *σ* = 0.01 S/m. The origin of coordinates is at the center of the rectangle and beginning of tunnel. Transmitter is placed at [-3.85,1,0] and both transmitter and receivers use vertical polarization with frequency 900 MHz. Maximum number of reflections is 20. Results show electric field computed with *RDN* and *LPR* simulations with different reception sphere radius (r) and either half isotropic (ISO) or sectorized (SEC) ray generation with different ray densities (rd). (a) Electric field for cross section at z = 100 m. (b) Electric field for cross section at z = 900 m.

As another scenario example, we discuss results for propagation on tunnels. Tunnels provide a demanding application scenario because the high number of reflections that effectively contribute to the received EM field [16]. To validate our results we have simulated a rectangular tunnel, for which approximate analytical results exist [16]. Simulations were done by placing all the receivers at the cross section, that is, the EM field is computed in parallel for 255 receivers, either with a single launch for isotropic generation (actually, only the forward hemisphere has been traced, since backward rays outside the tunnel do not contribute) or in multiple sequential launches with sectorized generation, each angular sector spans 10-degree in elevation by 10-degree in azimuth. In the latter case, the ray density is kept constant in all launches by setting the number of rays accordingly to the solid angle spanned by each sector. The analytical image-based [2] multi-modal solution has been computed with Matlab and plotted in Fig 7a together with the results computed by Opal with different simulation types: *RDN* and *LPR*. As can be seen, both simulation types match very closely the analytical results except at some particular positions, which highlights the limitations and differences between the methods. First, all of them depend on the sphere radius, which has to be set by the user and, unfortunately, there are no clear guidelines as to how select it for tunnels. In fact, for *LPR*, by examining the trace of the simulations it can be checked that the deviations at some positions are mainly due to extra rays hitting on the sphere which do not correspond to the theoretical images. On the contrary, for *RDN*, deviations result from not launching with ray density enough to properly average the contributions. However, past a certain radius, results degrade because in the average are included contributions with excessive angular dispersion. Therefore, keeping the sphere radius while increasing the ray density by using sectorized ray generation improves the accuracy, as shown in the figure. In fact, the advantage of *RDN* for this kind of simulation is that the achievable ray density is not limited by the available GPU memory.

At short and medium distances *LPR* can achieve a similar accuracy with much less ray density. But, at long distances, as shown in Fig 7b its performance degrades since the contributions kept by filtering are very sensitive to the sphere radius; and large radius cannot be used with high ray densities because of the GPU memory limitation. In that case, *RDN* is a better choice. As a summary, although both types of simulation methods provide very similar results in a wide number of scenario, *RDN* is usually more reliable when a large number of reflections are relevant and long distances are involved.

Regarding performance, it generally depends on the number of rays used per launch. Our simulations have been run on a server equipped with a Intel i9820 CPU, 32 GB RAM and 2 NVIDIA RTX 2080 Ti GPUs. In the single launch tests (half-isotropic) the computation time takes around 1 s, but this duration includes all the initial overhead required to create the acceleration other GPU context data. Looking at the multiple launch tests (sectorized), the duration for *RDN* with a constant density of 10^9^ rays/sr is 57.5 s, and since 324 sectors are computed, it means every launch takes on the order of 10^−1^ s. For *LPR* with a density of 4 ⋅ 10^7^ rays/sr it takes 3.6 s, which results in duration per launch on the order of 10^−2^ s.

#### Circular tunnel

**Fig 8:**
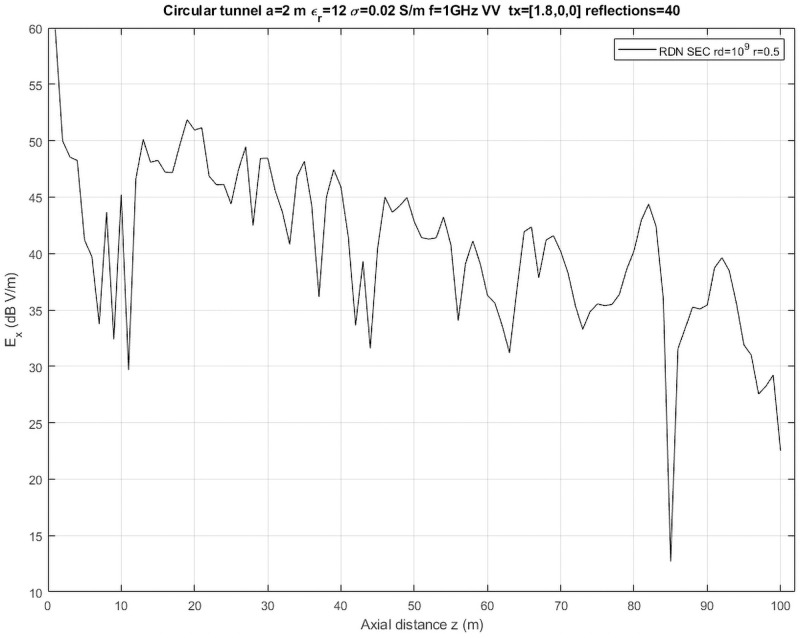
Circular tunnel. Received electric field for a circular tunnel of radius 2 m and length 1500 m and EM properties *ϵ*_*r*_ = 12 and *σ* = 0.02 S/m. Origin of coordinates is at center of circle at the beginning of tunnel. Transmitter is placed at [1.8, 0,0] m and transmits with vertical polarization at 1GHz. Sectorized ray generation (SEC): each sector spanning 10 degree of elevation by 10 degree of azimuth until the complete forward hemisphere is simulated. Maximum number of reflections is 40. Figure is to be compared with Fig 13 from [17].

The simulation of propagation with curved surfaces brings the following practical problems: (1) there are caustics, which are points where the divergence of rays [1] goes to infinity and means that the geometrical optic approximation is no longer valid at that point; and (2) conventional SBR methods need to filter multiple rays but there is no way to distinguish between different surfaces or scenario elements. Opal supports simulating propagation on curved surfaces with *RDN*, which overcomes the above problems. But still it is usually hard to obtain accurate results in some scenarios. As an example, we have simulated a circular tunnel (cylinder), for which an approximate analytical solution also exits [17]. We have placed 100 receivers separated 1 m along the longitudinal section of the tunnel and launch a sectorized simulation again with a constant density of 10^9^ rays/sr. The results are shown in Fig 8, which is to be compared with Fig 13 of [17]. This is a particularly hard scenario because of the multiple divergence and convergence of rays due to curvature and Opal is able to approximately reproduce them only with a very high number of reflections and ray density and a large reception radius even at short distances. At long distances, it is very difficult to obtain accurate results for these scenarios and it is necessary to resort to other types of simulation, usually based on physical optics, not implemented yet in Opal.

The performance, despite the increased complexity of the computations needed for curved surfaces, is only slightly worse and the above simulation required only 389 s to run, that is, 1.20 s per launch.

#### Edge diffraction

**Fig 9:**
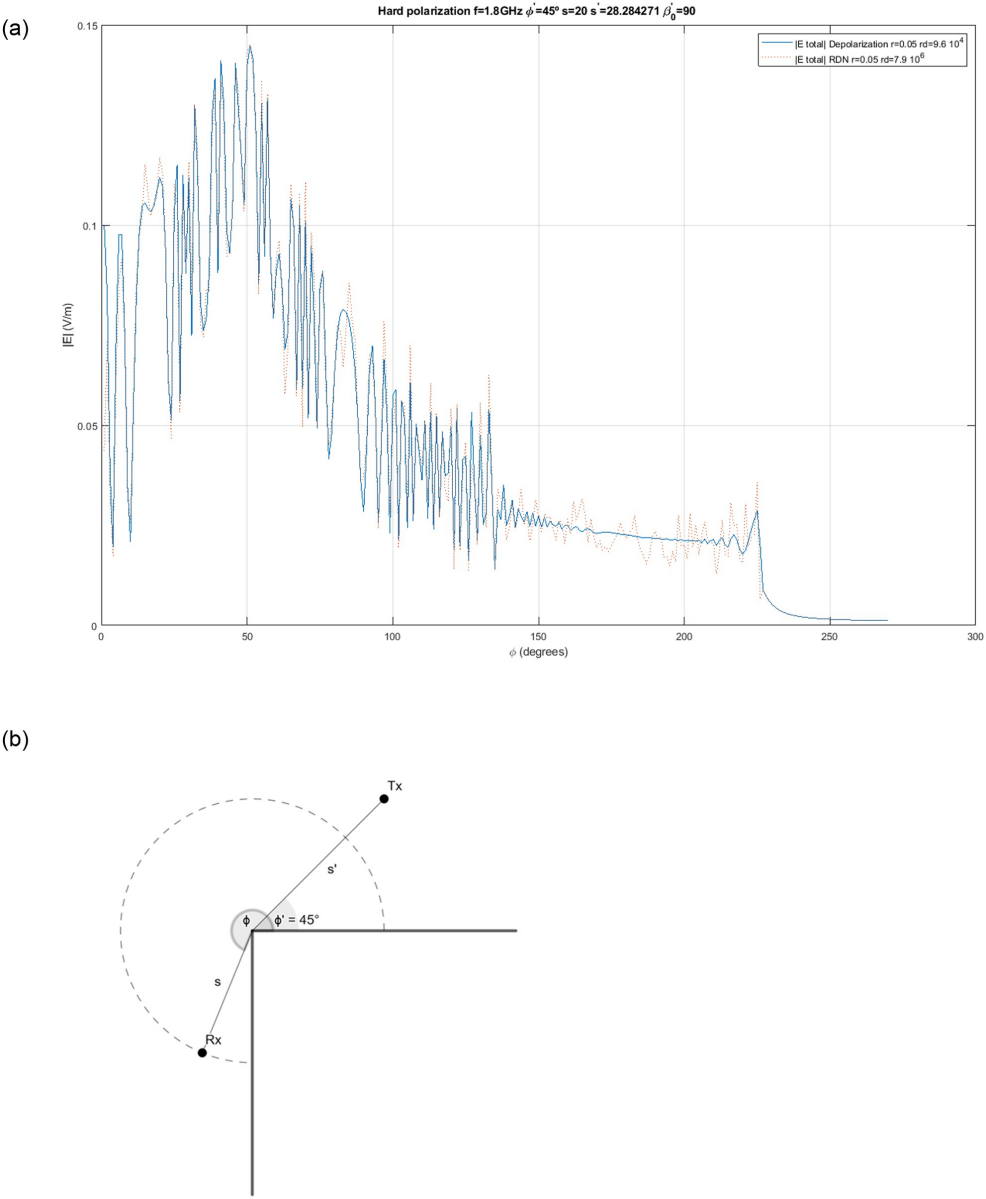
Straight edge diffraction. Magnitude of the total (direct, reflected, diffracted) received electric field at receiver points around a straight perfect conductor edge (square angle between faces, n = 1.5). Receivers are placed on a full circumference of radius s = 20 m around the edge at 1 degree steps. A transmitter at f = 1.8 GHz with vertical polarization is placed at an angle of 45 degrees from the edge closest face and $s^{\prime }=20\sqrt{(}2)$ m from the edge. (a) Total received field. (b) Geometry of the scenario.

In the examples so far, *RDN* seems to be superior to more conventional methods. But this is not always the case. In the last example we show a canonical problem: reflection and diffraction on a perfect conductor straight edge [1]. In the scenario, 270 receivers are placed around a square angle edge (n = 1.5) and an isotropic launch is executed. The results are shown in Fig 9. In this case, *LPR* obtains more accurate results because, even though the configured sphere radius make receivers capture additional contributions from direct rays, the filtering algorithm only keeps the hit closest to the receiver, whereas *RDN* does not and the ray density is not high enough to compensate for the deviations in the average.

#### Urban scenario

In the previous sections we validated our results again approximate (but very precise) analytical solutions. In this last section we validate against measurements done in a urban scenario. For this task, we have employed a set of measurements for the GSM band done by the authors more than ten years ago in the city of Cartagena. The measurement setup is described in detail in [18]. To reproduce the setup in Opal, we generated the corresponding area of the city of Cartagena (Spain) which includes 1067 buildings and 15082 diffraction edges, and placed 898 receivers as well as the base station antenna. We simulated up to 10 reflections with RDN plus single diffraction, using an isotropic launch with a ray density of 7.95 ⋅ 10^6^ rays/sr and sphere radius of 0.1. To account for position errors, we have replicated the simulation 10 times, displacing slighty the receivers by adding a random value uniformly distributed between 0 and 0.5 m to each dimension, and averaged the results.

**Fig 10:**
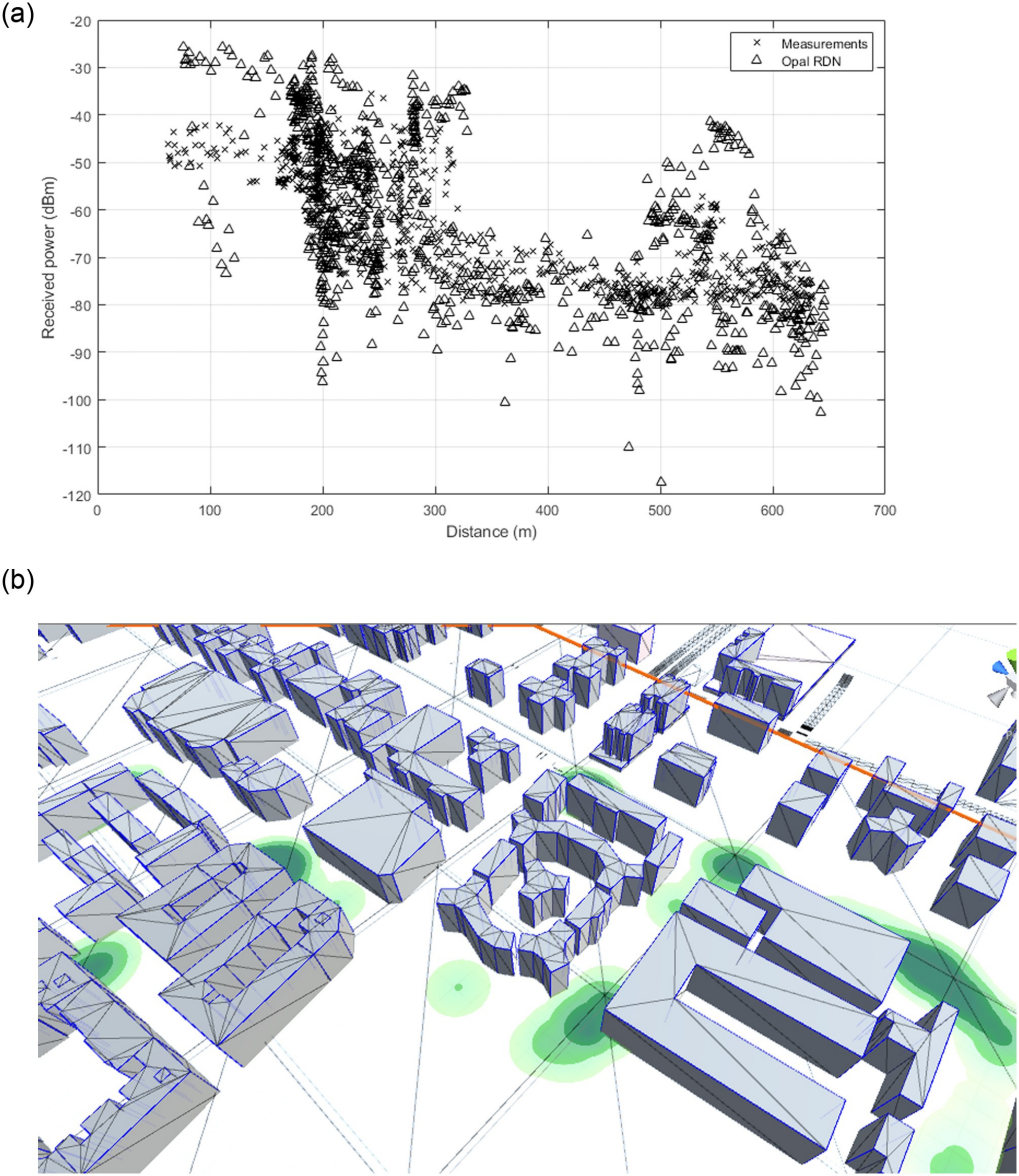
Comparison of measurements at 1.8 GHz and simulation in the city of Cartagena. The generated scenario includes 1067 buildings and 15082 diffraction edges. 898 receivers were simulated simutaneously. Each simulation point show the average of 10 replications. (a) Urban measurements. (b) Snapshot of a heatmap of the received EM field.

The results depicted in Fig 10(a) show good agreement between measurements and simulations, in spite of significant changes in the map due to recent constructions in the area, not present during the measurement campaign. Moreover, the average simulation time per replication is only 1.82 s with a standar deviation of 0.04 s, which also illustrates that urban simulations are less demanding in terms of required ray density and computational power than tunnel simulations. Finally, in Fig 10(b) we provide a snapshot from a heatmap generated from the received EM field strength to highlight the visualization capabilities of Opal.

## Discussion

Ray launching methods for propagation simulation go back several decades, but recent advances including the use of GPU are discussed in [2]. Even though they are more accurate that most of other techniques, their computational cost and difficult implementation due to lack of availability of efficient ray tracing libraries has been traditionally a barrier for their use. For this reason, a number of recent algorithms that improve the SBR basic mechanism by identifying optimal launch areas, such as ETRT [19] and MRLMA [20]. There are also hybrid models, such as GEMV^2^ [3], where a simplified geometrical representation of the objects, just the outlines of buildings, is used together with stochastic methods. The model is very specific, aimed at vehicular network simulations, but the results reproduce real propagation accurately at a reasonable cost. Our approach is more general. The most similar work to Opal is found in [6], where authors also use NVIDIA OptiX to perform the ray tracing, but their implementation is quite different as they log the ray traces and post-process the results to perform the EM computations. In addition, unlike Opal, the code is not freely available.

Propagation simulation by ray launching has usually been used for EM characterization of static environments, where the transmitter is kept fixed at a few positions [2], mainly because of a lack of tools for proper representation of dynamical 3D environments and low performance of the tracing process. On the contrary, ray tracing engines such as OptiX have been primarily designed to obtain high performance and quality rendering for interactive video games and, as such, they are optimized for real-time changing scenes. By leveraging them, Opal allows to dynamically add transceivers and other objects and can actually bring real-time changes to propagation simulators with acceptable accuracy and performance. Only authors of [6] evaluate the performance of ray launching with moving vehicles, and, to the best of our knowledge, Opal is the only, non-commercial, open source framework with this feature.

In the examples discussed in this paper we have highlighted some of the limitations of the current methods. To cope with them, Opal has been designed to facilitate its extension, in particular, with additional propagation effects and new simulation methods. Simulation of diffuse scattering is a propagation effect intended to be added in next releases. But there are also new promising methods such as [11], which uses bi-directional RT to collect information about wavefronts incident on an interaction surface. This method is suitable for GPU computation, so it can be seamlessly integrated in Opal, and avoids incorrect ray contributions due to the sphere radius, one of the major problems of SBR methods.

## Conclusion

In this paper we presented and described Opal, an EM propagation simulation tool implemented with ray-tracing on GPU, which is part of the Veneris network-traffic simulation framework. We have described its most relevant features and provided implementation details, especially of the simulation architecture which supports its extensions with new methods and additional propagation mechanisms, while highlighting the current limitations of the implemented methods. We have provided application examples, first to illustrate the flexibility that offers for automated scenario generation; and then to validate the simulation on demanding scenarios, comparing the results with analytical solutions, with further discussions on the trade-offs between approaches and its performance.

We believe Opal will become a valuable tool for general EM characterization as well as vehicular network research. As a standalone tool or as part of the broader Veneris framework, Opal provides a set of features rarely matched by any other similar open source tool that we know of. As discussed previously, the number of methods and proposals for propagation simulation is very large, but very rarely those tools are made freely available. Opal may help with this situation since it has been designed to be extended with new methods. Special care has also been taken to make it helpful for the user without knowledge of the internals, thanks to the intuitive interface of the Unity engine as well as features such as the web scenario generation tool. The goal is that it may be also useful as a purely academic tool, where students can graphically experiment with propagation effects.
